# Mapping spatial memory in teleosts: a new Frontier in neural logging techniques

**DOI:** 10.3389/fphys.2024.1499058

**Published:** 2024-11-06

**Authors:** Susumu Takahashi, Fumiya Sawatani, Kaoru Ide, Takaaki K. Abe, Takashi Kitagawa, Yuya Makiguchi

**Affiliations:** ^1^ Laboratory of Cognitive and Behavioral Neuroscience, Graduate School of Brain Science, Doshisha University, Kyotanabe, Japan; ^2^ College of Bioresource Science, Nihon University, Fujisawa, Kanagawa, Japan; ^3^ Graduate School of Frontier Sciences, The University of Tokyo, Chiba, Japan

**Keywords:** spatial memory, neural logging, teleosts, neurologgers, telencephalon

## Abstract

Recent advancements in microelectromechanical system technology have significantly enhanced our ability to monitor neuronal activity in free-swimming fish without disrupting their natural movement, thereby greatly improving the capabilities of neural logging using “neurologger” technology. In this review, we compiled the findings from studies applying neurologgers to teleost fish, emphasizing the discovery of various spatial-cognition cells in regions of the telencephalon analogous to the mammalian hippocampus that are deeply involved in spatial navigation. We detailed how different fish species, such as goldfish and salmonids, correlate their neural activity with environmental boundaries, head direction, speed, and other navigational cues for spatial memory and navigation strategies. We critically analyzed the similarities and differences in these mechanisms to provide insights into the evolutionary aspects of spatial cognition. We also identified gaps in current methodologies and suggest directions for future research, emphasizing the need for further exploration of spatial encoding in aquatic environments. The insights gained herein suggest the existence of a complex and evolutionarily conserved substrate for navigation and memory in vertebrates, highlighting the potential of neurologgers to expand our understanding of spatial cognition.

## 1 Introduction

The study of spatial cognition in teleost fish has seen substantial advancements due to the development of cutting-edge technologies using microelectromechanical systems (Ide and Takahashi, 2022). These technologies, referred to as “neurologgers,” enable researchers to record neuronal activity in free-swimming fish, providing a more accurate representation of their natural behavior. Zebrafish, a commonly used model organism among teleosts (Lieschke and Currie, 2007), offers various advantages for experimental manipulation, such as genetic tractability and transparency during the larval stage, which cannot yet be applied to them owing to their small size. Understanding spatial cognition in teleosts is crucial, as it sheds light on the fundamental neural mechanisms that support navigation and memory in vertebrates, a topic of significant interest in neurobiology. Recent studies have focused on the telencephalon of teleost fish, particularly in species like goldfish and salmonids (Vinepinsky et al., 2020; Takahashi et al., 2021), and have revealed the presence of various space-responsive cells. These include cells analogous to the mammalian head-direction and border cells, indicating a sophisticated system for spatial navigation. Previous research has highlighted the role of these cells in encoding spatial information; however, questions remain regarding the differences in spatial information representation across species and the impact of environmental factors.

This review aimed to synthesize findings from recent studies on space-responsive cells in the telencephalons of goldfish and salmonids. It sought to compare the similarities and differences in spatial information processing between species, exploring how these species use different environmental cues for navigation. This review also examined the evolutionary implications of these findings, particularly concerning the conservation of spatial cognition mechanisms across vertebrates. We focused on the recent research conducted using advanced neurologgers. Although the review provides a comprehensive overview of the neuronal substrates involved in spatial cognition, its scope was limited to goldfish and salmonids; it did not cover other teleost species or vertebrates in detail, acknowledging the need for further research to generalize these findings across a broader range of species. The central hypothesis of this review was that the neural mechanisms underlying spatial cognition in teleosts are evolutionarily conserved as well as highly adaptable, reflecting the specific ecological needs and environmental contexts of different species.

Understanding spatial cognition in teleosts has broader implications for the field of neurobiology, particularly in understanding how complex cognitive functions have evolved across vertebrates. Insights gained from studying these fish can improve our understanding of neuronal processes in other animal species. Furthermore, the methodological advancements discussed in this review, such as the use of neurologgers, have the potential to revolutionize the study of neuronal activity in freely behaving animals, offering new avenues for research in various fields of biology and neuroscience.

## 2 Neural substrates of spatial cognition in teleosts

The telencephalon of teleosts, particularly in species like goldfish and salmonids, has been identified as a crucial region for spatial navigation, as evidenced by studies linking brain lesions to impaired behavior (Rodriguez et al., 2002). This area contains a variety of space-responsive cells, including those resembling mammalian head-direction cells (Vinepinsky et al., 2020; Cohen et al., 2023). These findings underscore the complexity and sophistication of the spatial navigation systems in teleosts, similar to those found in mammals.

Extensive studies have demonstrated the presence of neurons that respond to specific environmental boundaries and head directions in goldfish. These neurons are believed to be critical for the cognitive map, allowing fish to orient themselves in their environment. For instance, head-direction cells in the lateral pallium of goldfish consistently fire when the head is oriented in a specific direction (Vinepinsky et al., 2020). This is akin to the function of the head-direction cells in mammals, which are essential for maintaining spatial orientation during navigation. Additionally, goldfish possess edge-encoding cells that become active when the fish are near environmental boundaries, such as the walls of a tank, similar to the border cells and boundary vector cells found in mammalian brains (Vinepinsky et al., 2020; Cohen et al., 2023). These cells likely provide a reference frame for spatial orientation, helping the fish navigate by maintaining an awareness of their position relative to environmental boundaries. Interestingly, some neurons in the goldfish pallium encode speed and velocity-vector cells that show increased firing rates correlated with the fish’s swimming speed and vector along the swimming direction, respectively (Vinepinsky et al., 2020). These cells integrate information about head direction and speed, which are crucial for calculating the fish’s trajectory and planning navigational strategies. Such integration indicates a complex neural network capable of supporting advanced navigational behaviors, including homing and exploration.

A notable study on free-swimming salmonids used wireless neurologgers to record neuronal activity in the telencephalon. This study revealed that these fish also possess head-direction cells that exhibit neuronal responses similar to those observed in goldfish and rodents (Takahashi et al., 2021). These findings suggest a conserved mechanism of heading orientation signals for spatial navigation across vertebrates. Recording neuronal activity using biotelemetry in naturalistic settings provides a more comprehensive understanding of how teleosts process spatial information. This approach allows researchers to observe neuronal activity in real-world environments, thereby providing more accurate insights into the neural mechanisms underlying spatial navigation.

**FIGURE 1:**
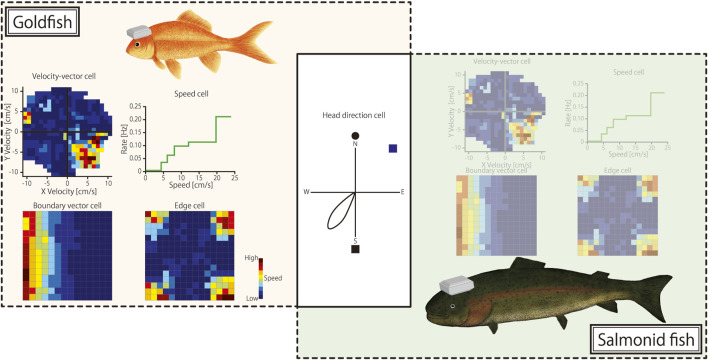
Spatial cognition cell types found in goldfish and salmonid fish. On the left, neural representations of spatial cognition cell types found in the telencephalon of goldfish are illustrated: (top left) velocity-vector cells that encode swimming velocity in two dimensions; (top right) speed cells with firing rates correlated with swimming speed; (bottom left) boundary-vector cells responding to environmental boundaries; and (bottom right) edge cells encoding edges of the environment. The central diagram shows a head-direction cell that fires when the fish’s head is oriented in a specific direction, a cell type found in both goldfish and salmonids. On the right, neuronal activity in the telencephalon of salmonid fish remains unclear for other spatial cognition cell types (denoted by shaded illustration), with only head-direction cells confirmed to be common between the two species.

A comparative analysis of goldfish and salmonids highlighted both similarities and differences in their spatial navigation mechanisms. Although both species share head-direction cells, the specific environmental cues they use may vary. This variation reflects adaptations to their respective habitats, with goldfish often relying more on visual cues (Broglio et al., 2003) and salmonids potentially using geomagnetic information for long-distance migration (Putman et al., 2013) (Figure 1). The absence of border cells in the pallium of salmonids may be due to their ethological needs and living environment. In studies on salmonids, the number of recorded cells and environmental variables are limited (Takahashi et al., 2021), which may be crucial for supporting or refuting this hypothesis in future research. In particular, because goldfish do not engage in long-distance migration, their response to changes in magnetic fields is a critical area for empirical study.

Despite these advancements, several challenges remain in the study of spatial cognition in teleosts. Most studies have been conducted in controlled laboratory environments, which may not fully capture the complexities of natural habitats. Future studies should explore how different environmental factors, such as water currents, salinity, changes in lighting, or underwater topography, affect the neural processing of spatial information. Additionally, understanding how teleosts encode three-dimensional space remains a significant challenge because their aquatic environments add complexity to spatial navigation. As goldfish are entirely freshwater fish and salmonids migrate between saltwater and freshwater, it is important to investigate, in the future, fish that exclusively inhabit oceanic environments, as we still do not fully understand what cues they use for spatial cognition. The continued development of wireless neurologgers and biotelemetry technologies is crucial for overcoming these challenges and enabling more detailed and extended studies in diverse and challenging environments. Overall, the exploration of space-responsive cells in teleosts has provided valuable insights into the neural substrates of spatial cognition, highlighting both the evolutionary conservation and adaptability of these systems across vertebrates.

## 3 Comparative analysis and evolutionary considerations

Comparative studies have highlighted similarities and differences between the spatial navigation systems of teleosts and mammals. For instance, while the hippocampal formation in mammals has been well-documented for its role in spatial memory (Wilson and McNaughton, 1993; Dickerson and Eichenbaum, 2010), homologous structures in teleosts, such as the lateral pallium, also play a significant role in spatial learning and memory. For example, the lateral pallium in goldfish has been implicated in the encoding of allocentric spatial strategies akin to the function of the hippocampus in mammals. Research involving lesion studies in goldfish has shown that damage to the lateral pallium impairs their ability to navigate using an allocentric strategy (Rodriguez et al., 2002), indicating its critical role in spatial cognition. Recent single-cell studies in teleost fish, particularly in goldfish and cichlids, provide additional evidence that the neuronal mechanisms supporting spatial navigation may be evolutionarily conserved. For example, mapping of the goldfish telencephalon using single-cell RNA sequencing and spatial transcriptomics revealed a hippocampal marker NEUROD6 expression scattered across the dorsolateral and dorsomedial regions (Tibi et al., 2023). Similarly, neurons in the subdivision of the dorsolateral pallium of cichlid fish share transcriptional profiling and neuroanatomy with mammalian hippocampus (Hegarty et al., 2024), offering further support for the hypothesis that spatial navigation mechanisms are conserved across vertebrates.

In mammals, spatial navigation relies heavily on the hippocampus and associated structures in which place cells (O’Keefe and Dostrovsky, 1971), grid cells (Hafting et al., 2005), border cells (Solstad et al., 2008), speed cells (Kropff et al., 2015) and head-direction cells (Taube et al., 1990) reside. These cells encode information regarding the animal’s location, direction, and distance traveled. Similarly, teleosts such as goldfish and zebrafish possess analogous neurons that respond to spatial cues, including edge-encoding cells that are activated near boundaries, and head-direction cells that maintain stable activity relative to the fish’s heading. Notably, recent research has identified place cells in the telencephalon of zebrafish (Yang et al., 2024), suggesting that teleosts share similar neural mechanisms for spatial cognition as mammals. These findings suggest that while the specific types of neurons and their functions can vary between species, the fundamental neural mechanisms supporting spatial navigation have been conserved across vertebrate evolution.

The use of geomagnetic cues in navigation is another area wherein significant differences emerge between aquatic and terrestrial vertebrates. For example, salmonids use geomagnetic cues for long-distance migration, a mechanism less prevalent in terrestrial mammals, which typically rely on visual and olfactory cues for navigation. This adaptation reflects the distinct environmental challenges faced by these species. The reliance on different sensory modalities for navigation in various environments underscores the versatility and adaptability of the spatial navigation systems in different taxa.

Furthermore, a comparative approach revealed that teleosts, such as goldfish, may lack certain features seen in mammalian spatial systems, such as theta oscillations (Cohen et al., 2023). These oscillations are prominent in mammalian hippocampal activity and are associated with navigation and memory encoding (Buzsáki, 2002). The absence of such rhythms in teleosts suggests that while the overall architecture of spatial cognition may be conserved, the specific neural dynamics can differ significantly from those in bats (Eliav et al., 2018).

Overall, these findings suggest that the neural mechanisms underlying spatial cognition have been conserved to some extent across vertebrate evolution. However, the specificity of how different species process spatial information varies, reflecting their adaptations to their respective environments. This comparative perspective not only highlights the evolutionary conservation of spatial navigation mechanisms, but also emphasizes the unique adaptations that different species have developed to navigate their specific ecological niches.

## 4 Methodological advances and future directions

The emergence of neurologgers has contributed significantly toward uncovering the neural bases of spatial cognition in teleosts. These tools enable high-resolution recording of neuronal activity in freely behaving animals and provide valuable insights into how these fish navigate complex environments. Methodological improvements are needed to overcome the current technological limitations. Although neurologgers have been revolutionary, challenges remain, such as the need for more robust waterproofing and longer battery life for extended recordings in underwater conditions. Moreover, developing smaller devices will allow researchers to study smaller species and younger individuals, broadening the scope of research on neural mechanisms in teleosts. Overall, these methodological advances and future directions highlight the potential for further discoveries regarding spatial cognition in teleosts. As we continue to refine our techniques and expand our understanding, we expect to gain deeper insights into the neural underpinnings of navigation and memory, not only in fish but also across the entire vertebrate lineage.

## 5 Discussion

Studies on spatial memory and navigation in teleosts highlight the remarkable complexity and sophistication of their neural systems. This body of research reveals that teleosts possess advanced spatial-cognition mechanisms, including space-responsive cells, such as head-direction cells, which are analogous to those found in mammals. These discoveries underscore the significant evolutionary conservation of spatial-cognition mechanisms across vertebrate lineages, suggesting that the ability to navigate and form an internal compass is a fundamental trait that may have evolved early in vertebrate history.

Teleosts, such as goldfish and salmonids, can use various environmental cues, including visual landmarks and geomagnetic fields, to navigate their environments. The presence of specialized neurons that respond to these cues indicates a highly developed neural substrate capable of processing complex spatial information. For instance, the discovery of head-direction cells in both goldfish and salmonids and their functional similarities to those in mammals points to a conserved neural architecture that supports spatial orientation and navigation across different species and ecological niches.

As research in this field advances, it is expected to further uncover the neural mechanisms that enable fish to navigate their aquatic habitats. This includes understanding how different types of space-responsive cells interact within broader neural networks, and how these networks integrate sensory information to support behaviors such as migration, homing, and exploration. The study of these systems in teleosts not only enhances our understanding of fish neurobiology, but also provides valuable insights into the evolution of cognitive functions in vertebrates.

The continued development and refinement of neurologgers is crucial for driving these discoveries. These tools have enabled researchers to record the neuronal activity in freely behaving animals, thereby offering a more accurate representation of how fish process spatial information in naturalistic settings. Future technological advancements may include more robust waterproofing, longer battery life, and device miniaturization, which will allow for extended and more detailed studies on smaller and younger fish.

Furthermore, future research should explore the neural encoding of three-dimensional space, given the unique challenges posed by the aquatic environments in which teleosts live. Understanding how these fish encode vertical and horizontal spatial information will provide deeper insights into the neural basis of three-dimensional navigation. The integration of neurologgers with other devices, such as depth sensors and accelerometers, in biotelemetry systems (Korpela et al., 2020; Otsuka et al., 2024; Tanigaki et al., 2024) may offer solutions to these challenges. Moreover, manipulating environmental variables, such as geomagnetic fields, in experimental settings can shed light on the specific neural circuits involved in processing these cues, further elucidating the mechanisms of spatial cognition, as the head-direction cells in the pallium of migratory birds prefer geomagnetic north (Takahashi et al., 2022).

In summary, research on spatial memory and navigation in teleost fish not only advances our knowledge of these fascinating animals, but also contributes to a broader understanding of the evolution of cognitive functions in vertebrates. As we continue to refine our methodologies and explore new technologies, we are likely to uncover new dimensions of the neural processes underlying spatial cognition. These findings have the potential to revolutionize our understanding of neurobiology and cognition across the animal kingdom, highlighting the shared and unique aspects of neural architecture and function that have evolved to support life in diverse environments.
